# Supplementary material for: Causal Relationship between Aging and Anorexia Nervosa: A White-Matter-Microstructure-Mediated Mendelian Randomization Analysis
Source: Biomedicines. 2024 Aug 16;12(8):1874. doi: 10.3390/biomedicines12081874 (PMC11351342; doi:10.3390/biomedicines12081874)
Supplement: Supplementary file 1 [file biomedicines-12-01874-s001.zip › Supplementary Figure/Figure S8/Leaveoneout plot of MR analysis for TL to ubm-b-1780.pdf]

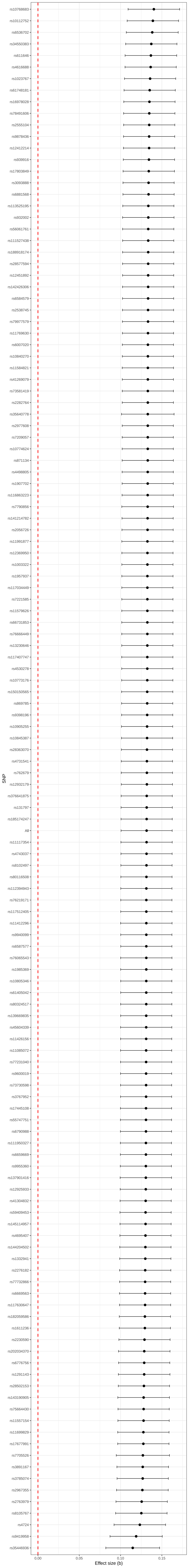

rs10768683
rs10112752
rs6536702
rs34550383
rs611646
rs4616688
rs1023767
rs61748181
rs16978028
rs78491606
rs2555104
rs9878436
rs12412214
rs939916
rs17803849
rs3093888
rs6881568
rs113525195
rs932002
rs56061761
rs111527438
rs188918174
rs28577594
rs12451892
rs142426306
rs6584579
rs2538745
rs79977579
rs11769630
rs6007020
rs10840270
rs11584821
rs41269079
rs73581419
rs2282764
rs35640778
rs2977608
rs7209057
rs10774624
rs871134
rs4498805
rs1907702
rs116863223
rs7790856
rs141214782
rs2056726
rs11991877
rs12369950
rs1003322
rs1957937
rs117034449
rs7221585
rs11579626
rs66731853
rs76666449
rs13230646
rs117407747
rs4530278
rs10773176
rs150150565
rs869785
rs9398196
rs10905255
rs10845387
rs28363070
rs4731541
rs762679
rs12932179
rs376641875
rs131797
rs185174247
A8
rs11117354
rs4743037
rs8102497
rs80116508
rs112394943
rs76219171
rs117512405
rs11412296
rs9940099
rs6587577
rs76065543
rs1985369
rs10805346
rs61405042
rs80324517
rs139669835
rs45604339
rs11426156
rs11085072
rs77231040
rs9600019
rs73730598
rs3767952
rs17445108
rs55747751
rs6790988
rs111050327
rs6659669
rs9955360
rs137901416
rs12925933
rs41304832
rs59409453
rs145114957
rs4695407
rs144204502
rs1332941
rs2276182
rs77732866
rs6669563
rs117630647
rs182059566
rs1611236
rs2230590
rs202034370
rs6776756
rs1291143
rs28502153
rs143190905
rs75664430
rs11557154
rs11699829
rs17677991
rs7705526
rs3891167
rs3785074
rs2967355
rs2763979
rs8105767
rs4724
rs9419958
rs35446936
SNP
0.00
0.05
0.10
0.15
Effect size (b)
